# Species-Specific Differences in Sperm Chromatin Decondensation Between Eutherian Mammals Underlie Distinct Lysis Requirements

**DOI:** 10.3389/fcell.2021.669182

**Published:** 2021-04-30

**Authors:** Jordi Ribas-Maynou, Estela Garcia-Bonavila, Carlos O. Hidalgo, Jaime Catalán, Jordi Miró, Marc Yeste

**Affiliations:** ^1^Biotechnology of Animal and Human Reproduction (TechnoSperm), Institute of Food and Agricultural Technology, University of Girona, Girona, Spain; ^2^Unit of Cell Biology, Department of Biology, Faculty of Sciences, University of Girona, Girona, Spain; ^3^Department of Animal Selection and Reproduction, Regional Agrifood Research and Development Service of Asturias (SERIDA), Gijón, Spain; ^4^Equine Reproduction Service, Department of Animal Medicine and Surgery, Faculty of Veterinary Medicine, Autonomous University of Barcelona, Bellaterra, Spain

**Keywords:** sperm, DNA condensation, DNA damage, protamine, mammals

## Abstract

Sperm present a highly particular DNA condensation that is acquired during their differentiation. Protamines are key elements for DNA condensation. However, whereas the presence of protamine 1 (P1) is conserved across mammalian species, that of protamine 2 (P2) has evolved differentially, existing only few species that use both protamines for sperm DNA condensation. In addition, altered P1/P2 ratios and alterations in the expression of P1 have previously been associated to infertility and DNA damage disorders. On the other hand, different methods evaluating DNA integrity, such as Sperm Chromatin Dispersion (SCD) and Comet tests, need a previous complete DNA decondensation to properly assess DNA breaks. Related with this, the present study aims to analyze the resilience of sperm DNA to decodensation in different eutherian mammals. Sperm samples from humans, horses, cattle, pigs and donkeys were used. Samples were embedded in low melting point agarose and treated with lysis solutions to induce DNA decondensation and formation of sperm haloes. The treatment consisted of three steps: (1) incubation in SDS + DTT for 30 min; (2) incubation in DTT + NaCl for 30 min; and (3) incubation in DTT + NaCl with or without proteinase K for a variable time of 0, 30, or 180 min. How incubation with the third lysis solution (with or without proteinase K) for 0, 30, and 180 min affected DNA decondensation was tested through analyzing core and halo diameters in 50 sperm per sample. Halo/core length ratio was used as an indicator of complete chromatin decondensation. While incubation time with the third lysis solution had no impact on halo/core length ratios in species having P1 and P2 (human, equine and donkey), DNA decondensation of pig and cattle sperm, which only present P1, significantly (*P* < 0.05) increased following incubation with the third lysis solution for 180 min. In addition, the inclusion of proteinase K was found to accelerate DNA decondensation. In conclusion, longer incubations in lysis solution including proteinase K lead to higher DNA decondensation in porcine and bovine sperm. This suggests that tests intended to analyze DNA damage, such as halo or Comet assays, require complete chromatin deprotamination to achieve high sensitivity in the detection of DNA breaks.

## Introduction

Different proteins encoded in nuclear and mitochondrial DNA allow cells to accomplish multiple functions to survive and proliferate. Nuclear chromatin consists of a complex of DNA and histone octamers. Histone octamers are formed by histones 2A, 2B, 3, and 4 (H2A, H2B, H3, and H4) and are separated with linker DNA of about 20–60 base pairs; each octamer condenses around 146 base pairs of DNA. The DNA-histone complex is highly supercoiled, forms chromatin fibers of 100–400 Kb in length and is organized in loop domains (Luger et al., 1997; Rao et al., 2014; Gibson et al., 2019). According to the models of chromatin organization, in each of these loops can one find a matrix attachment region (MAR) whose function is attaching chromatin to the nuclear matrix, a protein scaffold that supports nuclear organization (Ward, 2010) and contributes to the regulation of DNA replication and transcription in somatic cells, providing epigenetic plasticity for gene expression (Getzenberg, 1994; Linnemann et al., 2009; Ji et al., 2013; Narwade et al., 2019).

Spermatozoa are highly differentiated cells whose main function is the transport of genetic material to the oocyte through the female reproductive tract. To achieve this, millions of years of evolution have conferred mammalian sperm with highly specialized characteristics, such as the presence of flagellum, organization of mid-piece, and a very particular chromatin condensation and organization, as DNA is mainly condensed in protamines rather than in histones (Ward and Coffey, 1991; Wykes and Krawetz, 2003; Oliva, 2006). Organization of sperm chromatin changes during spermatogenesis, as histones are replaced by protamines through a series of events that, in some species such as humans, mouse, rat or sheep, involve transition nuclear proteins (Fuentes-Mascorro et al., 2000; Kierszenbaum, 2001; Hao et al., 2019). Condensation of sperm DNA through protamines protects genetic information from damaging agents (Zini and Libman, 2006), facilitating the delivery of an intact genetic material to the future embryo. This is very relevant as sperm DNA damage has been proposed as an important affectation that may underlie fertilization failure, poor embryo development, miscarriage and the production of unhealthy offspring (Makker et al., 2009; Lewis and Simon, 2010). These effects, however, are subject to the DNA damage repair capacity provided by the oocyte and, in this regard, different works described the activation of embryo checkpoints upon fertilization with damaged sperm (Adiga et al., 2007; Toyoshima, 2009). Also, a recent study in humans showed that ICSI cycles performed with sperm containing DNA damage depict lower embryo quality, pregnancy and implantation rates and higher miscarriage rates in older than in younger women (<36 years old) (Setti et al., 2021).

Protamines are arginine-rich proteins sized about half of the size of a typical histone and with highly positive charge, features that result in a tight binding to the nuclear sperm DNA, neutralizing the phosphodiester backbone (Balhorn, 1982). Protamine-DNA complex is responsible for the higher level of DNA condensation of spermatozoa compared to somatic cells; nuclear sperm DNA is known to be about six times more condensed than mitotic chromosomes, resulting in a nucleus with about 44 times less volume than that of a mouse liver cell (Ward and Coffey, 1991). Due to this DNA condensation, sperm chromatin is transcriptionally silent and, after fertilization, the replacement of protamines by histones is one of the main processes happening in the male pronuclear stage (Ajduk et al., 2006; Ward, 2010). In mammals, two different types of protamines (protamine 1 and protamine 2 family) are encoded in the genome. While protamine 1 is present in sperm from all species, protamine 2 family, formed by P2, P3, and P4 components, is only found in species such as humans, mice and horses (Fuentes-Mascorro et al., 2000; Corzett et al., 2002; Oliva, 2006; Gosálvez et al., 2011). Whereas protamine 1 is synthesized as a mature protein, those belonging to protamine 2 family (mainly P2, P3, and P4 are present in a minor percentage) need post-translational proteolysis prior to conversion into the mature form (Oliva, 2006). As Protamine 2 family proteins have never been found to compact sperm DNA alone, they appear not to be capable of condensing sperm chromatin without protamine 1 (Corzett et al., 2002). Alterations in the expression of protamine 1 have been shown to be linked to sperm morphology anomalies and premature DNA condensation during sperm differentiation (Peschon et al., 1989; Lee et al., 1995); alterations in protamine 2 content lead to male infertility and DNA damage (Aoki et al., 2005; Nanassy et al., 2011), as confirmed by *Prm2*^±^ knockouts (Oliva and Dixon, 1991; Cho et al., 2001).

The currently accepted model for chromatin organization in mammalian sperm purports that protamine-DNA complex forms toroidal structures, which condense about 48 kb of DNA, and are estimated to measure between 60 and 100 nm in diameter and 20 nm in thickness (Brewer et al., 1999, 2003; Miller et al., 2010). These toroidal structures are stabilized by disulfide covalent bonds between neighbor protamines, and are linked through uncoiled linker DNA in toroid linker regions (TLR). The function of TLR is to attach the chromatin to the nuclear matrix and is thus supposed to contain matrix attachment regions (MAR; Ward, 2010). Toroid linker regions are assumed to be condensed in histones containing complex epigenetic information that may play a crucial role in the very early zygote by influencing gene expression after fertilization and during early embryo development (Jung et al., 2017; Yoshida et al., 2018). In the context of sperm chromatin, localization of histone regions in gene-rich or gene-poor DNA is a subject of much debate among scientists, as different methodologies have been shown to lead to opposite results (Yamaguchi et al., 2018). Moreover, it has been demonstrated that different species may have different percentages of histone-condensed DNA, ranging between 3 and 15% in humans, between 1 and 15% in mouse or even at higher rates (like 50%) in some marsupials (Fuentes-Mascorro et al., 2000; Hammoud et al., 2009; Miller et al., 2010; Samans et al., 2014). Despite the fact that the toroidal DNA and the linker DNA coupled to the nuclear matrix constitute the main unit of chromatin structure, evidences obtained through freeze-fracture, circular dichroism and atomic force microscopy support the presence of a higher superstructure of sperm chromatin, composed by parallel stacks of lamellar sheets oriented to the nuclear long axis in bulls, bucks and men (Koehler et al., 1983; Fuentes-Mascorro et al., 2000). Also, other evidences support a nodular chromatin sub-organization, sized between 100 and 300 nm, in mouse, human, cattle and rat sperm (Allen et al., 1993; Haaf and Ward, 1995). However, the presence of smaller nucleosome-like particles is not discarded, especially in the nuclear periphery (Allen et al., 1996). Regardless of chromatin superorganization, different studies also reported a chromosomal region-specific organization, since centromeres were found to be positioned in the most internal parts of the sperm nucleus, whereas telomeres that remain condensed in histones appeared to attach to the internal nuclear membrane, positioned in the external parts of the nucleus (Zalensky et al., 1997; Mudrak et al., 2005; Zalensky and Zalenskaya, 2007). Moreover, other authors showed that chromosomal territories exist in the sperm nucleus, leading to a precise organization of chromosomal DNA (Luetjens et al., 1999; Zalenskaya and Zalensky, 2004; Foster et al., 2005).

While the high degree of sperm DNA condensation requires protamines to be replaced by histones upon fertilization, it also represents a challenge for the *in vitro* study of sperm chromatin. In this sense, previous studies tested the *in vivo* sperm DNA decondensation of different animal species after microinjection of hamster oocytes; whereas nuclei of human, mouse, chinchilla and hamster sperm were properly decondensed, this was not the case of cattle or rat sperm (Perreault et al., 1988). The same study showed that, while DTT and SDS led to sperm DNA decondensation, incubation with these two agents needed to be 10 times longer in cattle than in human sperm to achieve similar levels of decondensation (Perreault et al., 1988). Nowadays, NaCl is known to be strictly necessary to remove protamines from DNA, a required step to achieve complete DNA decondensation and form sperm haloes of decondensed chromatin, leaving TLR attached to the nuclear matrix (Mohar et al., 2002; Sotolongo et al., 2003).

Different methods require complete sperm DNA decondensation to evaluate DNA breaks. DNA fragmentation assays, such as the SCD test, rely on the differential chromatin decondensation of spermatozoa containing DNA breaks compared to those with non-damaged DNA (Fernández et al., 2003; Cortés-Gutiérrez et al., 2009). Likewise, the Comet assay requires complete DNA decondensation for migration of fragments and the subsequent formation of the Comet tail, and some protocols include incubations with proteinase K overnight (Simon et al., 2014; Ribas-Maynou and Benet, 2019). Even the TUNEL assay has been proposed to display higher sensitivity following mild decondensation with DTT (Mitchell et al., 2011). Decondensation of sperm chromatin is therefore applied to multiple species, with several studies linking DNA breaks to fertility disorders (Pérez-Llano et al., 2010; Gosálvez et al., 2011; Esteves et al., 2020; Ribas-Maynou et al., 2020).

Against this background, this study aimed at comparing the decondensation capacity of sperm DNA between different species, some described to contain both protamine 1 and protamine 2, and others known to have protamine 1 only.

## Materials and Methods

### Reagents

Unless stated, all reagents used for this study were purchased from Sigma-Aldrich (St. Louis, MO, United States).

### Sperm Sample Obtaining and Cryopreservation

#### Human Sperm

Five human semen samples were obtained from healthy volunteers that were recruited by University of Girona, Spain. All donors signed an informed consent before providing their biological samples, and the study was approved by the Ethics Committee, Josep Trueta Hospital, Spain (Reference PTI-HUMA 10012018) thus complying with the Helsinki declaration for the research with human specimens. All men provided a semen sample through ejaculation in a sterile cup. This ejaculation was maintained at 37°C for 30 min and was subsequently cryopreserved.

Cryopreservation was performed using Test Yolk Buffer (TYB, 14% glycerol, 30% egg yolk, 1.98% glucose, 1.72% sodium citrate, and 2% glycine, pH 7.4). Briefly, each semen sample was gently mixed 1:1 (v:v) with TYB, poured into a 0.5-mL cryotube, and cooled down in an isopropyl alcohol bath placed within a −80°C freezer for 12 h. Afterward, cryotubes were transferred to liquid nitrogen. Samples were thawed by submerging cryotubes in a water bath at 37°C, and then diluted with PBS (1:3 dilution), added drop-by-drop in order to avoid osmotic shock.

#### Pig Sperm

Researchers did not manipulate animals, but semen samples were rather purchased from a local farm (Servicios Genéticos Porcinos, S.L.; Roda de Ter, Spain), which provided commercial, standard doses (90 mL) intended to artificial insemination (AI). A total of five semen samples were obtained from five sexually mature Piétrain boars of proven fertility. Ejaculates were collected using the gloved-hand method, and the sperm rich fraction was diluted to 3.3 × 10^7^ spermatozoa/mL with a long-term commercial extender (Vitasem LD; Magapor SL, Zaragoza, Spain).

Cryopreservation was performed using the following protocol. First, 50-mL aliquots were centrifuged at 2,400 × g and 15°C for 3 min, and sperm suspensions were diluted in β-lactose-egg yolk (LEY) medium (80% v:v lactose, 20% egg yolk) to a final concentration of 1.5 × 10^9^ sperm/mL. Thereafter, all aliquots were cooled down to a final temperature of 5°C, at a rate of −0.1°C/min. Then, samples were diluted to a final concentration of 1 × 10^9^ sperm/mL in LEYGO medium, which consisted of LEY medium supplemented with 6% v:v glycerol and 1.5% Orvus ES Paste (Equex STM; Nova Chemical Sales Inc., Scituate, MA, United States). Finally, samples were packed into 0.5-mL plastic straws (Minitüb, Tiefenbach, Germany), and a programmable controlled-rate freezer (Ice-Cube, Minitüb) was used to cryopreserve samples at the following cooling rates: 6°C/min from 5 to –5°C (100 s); –39.82°C/min from –5 to –80°C (113 s); holt at –80°C for 30 s; and cooled at –60°C/min from –80 to –150°C (70 s). Samples were finally plunged into liquid nitrogen.

Samples were thawed through submerging a 0.5-mL straw in a 38 °C water bath for 15 s, with shaking. The entire volume of the straw was diluted in three volumes (1:3, v:v) of pre-warmed Beltsville Thawing Solution (BTS).

#### Horse Sperm

Five cryopreserved straws from five separate animals were used. All stallions were donors of proven fertility, and were housed at the Equine Reproduction Service, Autonomous University of Barcelona (Spain), which is a European Union approved equine semen collection center (Authorization code: ES09RS01E). Semen samples were obtained using a Hannover-type artificial vagina (MiniTüb, Tiefenbach, Germany) that contains a nylon mesh filter to separate the ejaculate gel fraction. The obtained ejaculates were directly diluted (1:5, v:v) in previously warmed (37°C) Kenney buffer (49% glucose, w:v; 24% powdered milk, w:v).

Cryopreservation was performed according to the following protocol: first, samples were centrifuged at 600 × g and 20°C for 15 min. Then, pellets were resuspended in BotuCRYO commercial extender (Botupharma, Botucatu, Brazil) to reach a concentration of 2 × 10^6^ viable sperm/mL. Samples were then packaged into 0.5-mL straws and frozen in a programmable freezer (Ice-Cube) as follows: (i) −0.25°C/min from 20 to 5°C (60 min); (ii) −4.75°C/min from 5 to −90°C (20 min); and (iii) −11.11°C/min from −90 to −120°C (2.7 min). Straws were finally plunged into liquid nitrogen for storage.

Thawing was performed by submerging straws in a water bath at 38°C for 20 s. The volume of each straw was diluted in three volumes of pre-warmed Kenney medium (1:3, v:v).

#### Donkey Sperm

Five semen samples coming from five different jackasses of proven fertility were used. Animals were located at the Equine Reproduction Service, Autonomous University of Barcelona (Bellaterra, Spain). Semen samples were obtained using a Hannover artificial vagina (Minitüb) with a nylon mesh filter to remove the gel fraction. Immediately after collection, semen was mixed with Kenney extender (1:5, v:v) at 37°C.

For cryopreservation, the following protocol was used: first, diluted semen was centrifuged at 600 × g and 20°C for 15 min, and pellets were resuspended in 2 mL of INRA-freeze freezing extender (INRA, Paris, France). Then, concentration and motility were evaluated, and samples were diluted again to reach a final concentration of 2 × 10^6^ viable sperm/mL. Following this, sperm were packaged into 0.5-mL straws and frozen with a programmable freezer (Ice-Cube, Minitüb) with the following cooling rates: (i) from 20 to 5°C at a rate of −0.25°C/min; (ii) from 5 to −90°C at a rate of −4.75°C/min; and (iii) from −90 to −120°C at a rate of −11.11°C/min. Finally, straws were plunged into liquid nitrogen and stored.

For thawing, straws were submerged in a water bath at 38°C for 20 s, and the content was diluted with three volumes of in 1.5 mL of Kenney medium at 37°C per straw.

#### Cattle Sperm

Five separate samples from five healthy and sexually mature Holstein bulls were used. All animals were kept in an artificial insemination center (Cenero-Asturias, Gijón, Spain), and ejaculates were collected through an artificial vagina. Collected semen was diluted in Bioxcell extender reaching a concentration of 92 × 10^6^ sperm/mL. Then, samples were cooled down to 4°C at a rate of −0.2°C/min, and after 3 h of equilibration, extended semen was packaged into 0.25-mL straws containing 23 × 10^6^ sperm/straw.

Cryopreservation was performed in a programmable freezer (Digit-cool; IMV Technologies, L’Aigle, France) following the standard curve for bovine sperm: −5°C/min from 4 to −10°C, −40°C/min from −10 to −100°C, and −20°C/min from −100 to −140°C. Thawing was performed by submerging straws in a water bath at 38°C for 40 s, with shaking.

### Halo Formation

Halo formation was induced following the same protocol for all species. First, samples were diluted in phosphate-buffered saline (PBS) to reach a final concentration of 1 × 10^6^ sperm/mL. Meanwhile, an aliquot of low melting point agarose at 1% was melted at 70°C for 10 min and warmed to 37.5°C for further 10 min. Next, the sample and low melting point agarose were mixed at a 1:2 (v:v) ratio, obtaining a final agarose concentration of 0.66%. A drop of 6 μL of the mixture was then dispensed onto an agarose pre-treated slide, covered with a 8-mm circular coverslip, and allowed to jellify on a metal cold plate at 4°C for 5 min. Afterward, the coverslip was gently removed and the slide was incubated in a first lysis solution (0.8 M Tris-HCl, 0.8 M DTT, 1% SDS; pH = 7.5) at room temperature for 30 min. Following this, slides were incubated in a second lysis solution (0.4 M Tris-HCl, 0.4 M DTT, 50 mM EDTA, 2 M NaCl, 1% Tween20; pH = 7.5) at room temperature for 30 min. At this point, slides were differentially incubated with a third lysis solution that contained proteinase K (0.4 M Tris-HCl, 0.4 M DTT, 50 mM EDTA, 2 M NaCl, 1% Tween20, 100 μg/mL proteinase K; pH = 7.5). Incubation with this third lysis solution was also performed at room temperature for 0, 30, or 180 min. To assess the effect of incubating pig and cattle sperm with proteinase K, decondensation with a third lysis solution that did not contain proteinase K (0.4 M Tris-HCl, 0.4 M DTT, 50 mM EDTA, 2 M NaCl, 1% Tween20, 100 μg/mL proteinase K; pH = 7.5) was also performed for 0, 30, or 180 min. After all lysis steps, slides were washed with a neutral pH solution (0.4 M Tris-HCl, pH 7.5) for 5 min, and dehydrated in ethanol series (70, 90, and 100%; 2 min/step).

### Assessment of DNA Decondensation

**FIGURE 1:**
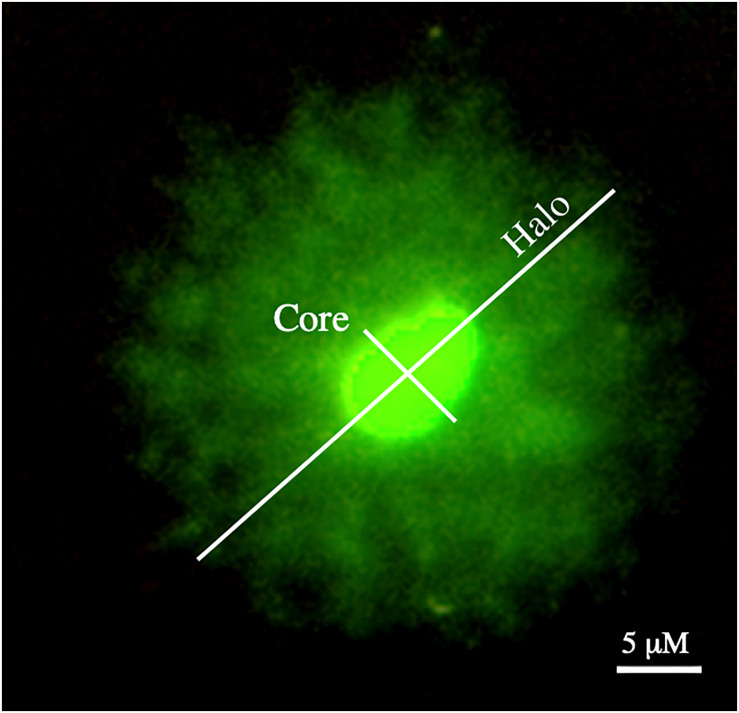
Measurement of core and halo in sperm cells with decondensed chromatin.

Halo and core diameters are used as DNA decondensation indicators. In order to obtain both measures, samples were labeled with Safeview DNA stain (NBS Biologicals, Huntingdon, United Kingdom) prior to evaluation under a Zeiss Imager Z1 epifluorescence microscope (Carl Zeiss AG, Oberkochen, Germany). Captures at 1,000× magnification of at least 50 cells per experimental condition were obtained using Axiovision 4.6 software (Carl Zeiss AG, Oberkochen, Germany). In these captures, exposure time was adapted to correctly differentiate haloes from core sperm heads. From these pictures, diameters of the core and the halo were measured as indicated in Figure 1, using the Image J measurement tool. These diameters were obtained in duplicate, and mean values were calculated for each sperm cell. In order to normalize data, a scale bar was used to obtain the correspondence of pixels to μm at the resolution used (1,388 × 1,040 pixels).

### Staining of Nuclear DNA in Non-decondensed Sperm Cells

In order to measure the size of nuclear DNA, a sperm sample from each species was smeared onto a glass slide, permeabilized with PBS containing 0.25% Triton X-100 for 2 min, and stained with Safeview DNA stain. Measures of nuclear core were made following the same procedure depicted in Figure 1.

### Statistics

Data were analyzed using the Statistics Package for the Social Sciences software (IBM SPSS ver. 25; IBM Corp., Armonk, NY, United States). Graphs were drawn using GraphPad Prism Software (GraphPad; San Diego, CA, United States). Once data were recorded and compiled, normal distribution and homogeneity of variances were checked through Shapiro-Wilk and Levene tests. Since, even after linear transformation [log(x), arcsine √x, √x], data did not fit with parametric assumptions, Scheirer-Ray-Hare and Mann-Whitney tests were run. A significance level of 95% for the confidence interval was established to consider statistically significant differences.

## Results

**FIGURE 2:**
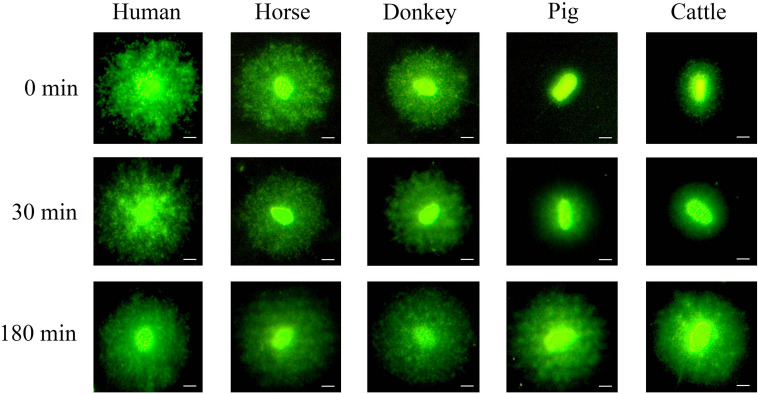
Decondensation of sperm DNA through the formation of sperm haloes. It is worth noting that porcine and bovine sperm presented higher DNA decondensation with increasing incubation times over the third lysis step containing proteinase K. In contrast, no significant changes in human, horse and donkey sperm were observed. Scale bar: 5 μm.

Figure 2 shows the formation of sperm haloes in different species, following different incubation times with proteinase K (third lysis step). The application of the third lysis step containing proteinase K had a differential effect on the separate species studied. Whilst human, horse and donkey sperm did not present higher decondensation when incubated with proteinase K for a longer period (third lysis step), pig and cattle sperm showed higher DNA decondensation after incubation with proteinase K and exhibited larger sperm haloes.

### Lysis Solutions Used for Halo Formation Induce Nuclear Core Decondensation

Following DNA staining of sperm spreads/smears, dimensions of nuclei were found to be: 4.14 μm ± 0.41 μm (mean ± standard deviation) for human sperm, 3.62 μm ± 0.55 μm for horse sperm, 3.89 μm ± 0.52 μm for donkey sperm, 4.43 μm ± 0.34 μm for pig sperm, and 4.80 μm ± 0.34 μm for cattle sperm. While treatment with lysis solutions that did not contain proteinase K led to chromatin decondensation of sperm core, the extent of that decondensation differed between species. In effect, while, after this step, the cores of human, horse and donkey sperm were 7.11 μm ± 0.96 μm, 5.67 μm ± 0.55 μm, and 5.83 ± 0.35, respectively, they were found to measure 4.98 μm ± 0.50 μm in pig sperm and 4.34 μm ± 0.79 μm in cattle sperm. In spite of this, this increase in the diameter of the core, which indicated DNA decondensation, was statistically significant in all cases (human, *P* < 0.001; horse, *P* < 0.001; donkey, *P* < 0.001; pig, *P* < 0.001; and cattle, *P* = 0.004).

Moreover, nuclear core also showed an increase in decondensation associated to the additional treatment of sperm cells with a lysis solution containing proteinase K (Table 1). This increase was statistically significant in horse, donkey, porcine and bovine sperm when comparing 180 min of incubation with 0 min of incubation (*P* < 0.05); and in human, porcine and bovine sperm when comparing 180 min of incubation with 30 min of incubation (*P* < 0.05).

**TABLE 1:** Effect of the third lysis solution containing proteinase K on halo and core diameter in different species.

	Incubation time (min)	Human	Horse	Donkey	Pig	Cattle
Core with (μm)	0	7.11 ± 0.96	5.67 ± 0.55	5.83 ± 0.35	4.98 ± 0.50	4.34 ± 0.79
	30	6.62 ± 1.17	6.92 ± 0.58^a^	6.32 ± 0.64	5.28 ± 0.39	6.05 ± 0.58^a^
	180	8.19 ± 0.98^b^	6.96 ± 1.60^a^	7.15 ± 0.79^a^	6.37 ± 0.61^a,b^	7.16 ± 1.01^a,b^
Halo + core diameter (μm)	0	32.45 ± 0.86	22.91 ± 1.46	25.18 ± 3.67	8.62 ± 1.04	10.36 ± 3.71
	30	27.74 ± 2.54^a^	30.28 ± 1.39^a^	24.55 ± 2.78	15.87 ± 1.32^a^	20.15 ± 3.22^a^
	180	37.76 ± 1.46^a,b^	30.69 ± 2.41^a^	29.32 ± 2.82^b^	25.81 ± 1.53^a,b^	29.17 ± 2.50^a,b^
Halo/Core ratio	0	4.68 ± 0.51	4.15 ± 0.47	4.40 ± 0.64	1.77 ± 0.20	2.40 ± 0.40
	30	4.32 ± 0.51	4.57 ± 0.53	4.00 ± 0.37	3.05 ± 0.14^a^	3.36 ± 0.22^a^
	180	4.77 ± 0.64	4.68 ± 0.63	4.17 ± 0.19	4.13 ± 0.39^a,b^	4.19 ± 0.38^a,b^

*^*a*^Statistical differences with respect to incubation with proteinase K for 0 min (P<0.05). ^*b*^Statistical differences with respect to incubation with proteinase K for 30 min (P<0.05).*

### Prolonged Lysis Incubation Contributes to Higher Efficiency in Protamine Removal

Table 1 shows the effect of incubation in the third lysis solution containing proteinase K on halo diameter. We observed that exposure to proteinase K led to higher halo diameters in all species. In effect, there was a significant (*P* < 0.05) increase in halo diameters of human, horse, pig and cattle sperm, when 0 and 180 min of incubation were compared. Furthermore, 180-min incubations compared to their 30-min counterparts also showed a significant increase in halo diameters of human, donkey, pig and cattle sperm (*P* < 0.05). However, and in spite of these significant differences, the extent of the increase of halo diameter in porcine and bovine sperm was much higher (Table 1).

### The Halo/Core Ratio Reflects the Decondensation Status of a Sperm DNA

**FIGURE 3:**
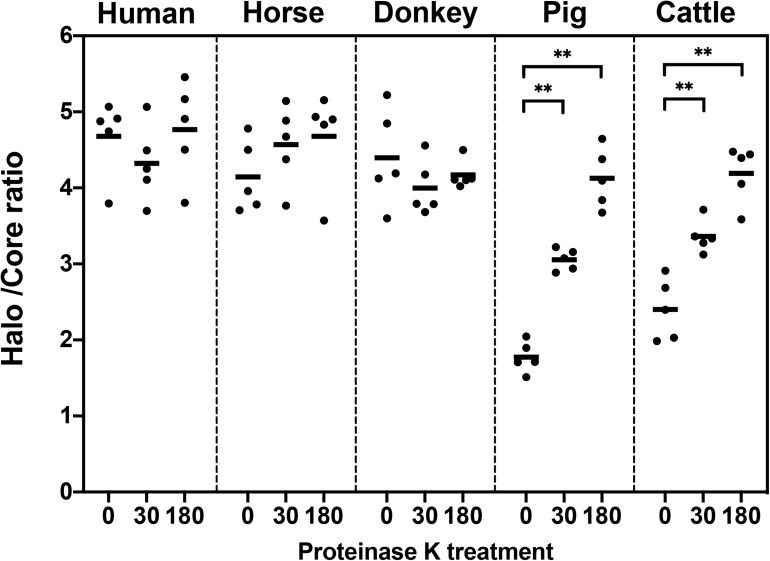
Halo/core ratio in relation to proteinase K treatment (0, 30, or 180 min). ^∗∗^Statistically significant differences (*P* < 0.05).

Since both sperm haloes and cores showed an increased size associated to proteinase K lysis treatment, the relationship between halo size and core size appeared to be an indicator of sperm DNA decondensation. Figure 3 and Table 1 show the halo/core ratio for all species involved in this study. Interestingly, while human, horse and donkey sperm did not exhibit an increase in halo/core ratios associated to longer proteinase k treatments (*P* > 0.05), these ratios augmented in porcine and bovine sperm (0 min vs. 30 min; 30 min vs. 180 min; *P* < 0.05).

### The Inclusion of Proteinase K in the Third Lysis Solution Accelerates DNA Decondensation

**FIGURE 4:**
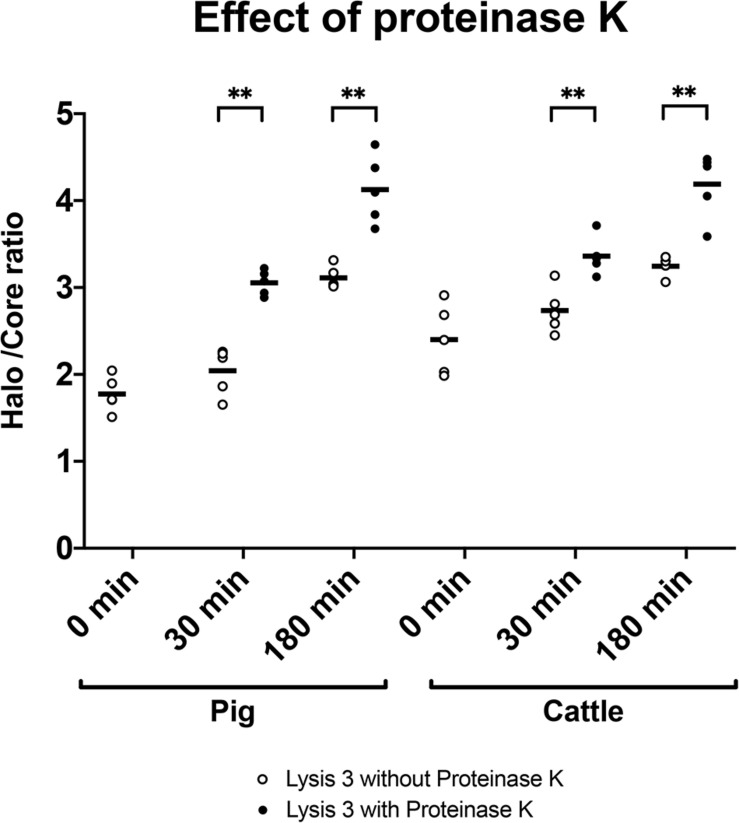
Halo/core ratios for incubations (0, 30, or 180 min) of pig and cattle sperm in the third lysis solution without (empty dots) or with proteinase K (filled dots). ^∗∗^Statistically significant differences (*P* < 0.001).

To determine the importance of including proteinase K in the third lysis solution, we tested DNA decondensation in pig and cattle sperm after incubation with the same lysis solution without proteinase K for 30 and 180 min. Table 2 shows core diameter, halo diameter and halo/core ratio after such incubations. Figure 4 depicts halo/core ratio as a measure of DNA decondensation. When proteinase K was included, halo diameter significantly increased both in pig and cattle sperm after 30 and 180 min of incubation (*P* < 0.001). Similarly, all tested conditions also augmented halo/core ratios in both species (*P* < 0.001).

**TABLE 2:** DNA decondensation of pig and cattle sperm after incubation in the third lysis solution with or without proteinase K.

		Pig		Cattle	
	Incubation time (min)	− Proteinase K	+ Proteinase K	− Proteinase K	+ Proteinase K
Core with (μm)	0	4.85 ± 0.85		4.22 ± 0.95	
	30	5.30 ± 0.57	5.20 ± 0.81	6.01 ± 1.03	5.95 ± 0.91
	180	4.80 ± 0.53	6.24 ± 0.91**	6.63 ± 0.68	7.02 ± 1.38*
Halo + core diameter (μm)	0	8.37 ± 8.37		9.87 ± 9.87	
	30	10.99 ± 10.99	15.49 ± 15.49**	16.20 ± 16.20	19.54 ± 19.54**
	180	14.92 ± 14.92	25.63 ± 25.63**	21.40 ± 21.40	28.83 ± 28.83**
Halo/Core ratio	0	1.76 ± 0.35		2.38 ± 0.62	
	30	2.10 ± 0.37	3.04 ± 0.54**	2.75 ± 0.51	3.32 ± 0.50**
	180	3.13 ± 0.45	4.19 ± 0.72**	3.26 ± 0.45	4.23 ± 0.74**

*Statistical differences with respect to incubation without proteinase K (P<0.05). **Statistical differences with respect to incubation without proteinase K (P<0.001).

## Discussion

In the present study, we observed complete decondensation of sperm DNA when samples were exposed to a lysis solution containing proteinase K for a long period (up to 180 min). We found inter-specific differences regarding sperm chromatin decondensation following that lysis treatment since, while human, horse and donkey sperm did not effectively augment their DNA decondensation, pig and cattle sperm achieved a 2.3- and 1.75-fold increase, respectively (based on halo/core ratios). Moreover, in an additional experiment, we could establish that proteinase K accelerates DNA decondensation in pig and cattle sperm.

A high DNA condensation up to almost crystalline state is a key feature that is only present in paternal germ cells and allows them to transport the male genome through the female tract to reach the oocyte. This highly condensed state helps in the protection of sperm cells from genotoxic damage to which they are exposed (Adiga et al., 2007; Marchetti et al., 2015). Due to their transcriptional silence and the loss of most of their cytoplasmic proteins, sperm cells are devoid of DNA repair systems; however, they are continuously exposed to exogenous and endogenous DNA damage, caused mainly through oxidative stress (Aitken and De Iuliis, 2007). Not only are reactive oxygen species produced by sperm mitochondria through oxidative phosphorylation, which is essential for ATP generation (Camello-Almaraz et al., 2006), but can also result from the presence of immature sperm, leucocytes, and other exogenous effectors, such as pollutants, heat stress, infections, radiations, or other toxicants (Agarwal et al., 2008; Ribas-Maynou et al., 2020).

Different methods that indirectly evaluate DNA integrity are based on the differential decondensation of sperm cells that contain DNA breaks. In this context, *in vitro* DNA decondensation is required in order for external and internal chromatin to be accessible. As previous studies showed, methods evaluating sperm DNA integrity differ in their sensitivity to detect the actual DNA damage (Ribas-Maynou et al., 2013; Simon et al., 2014). In this regard, achieving complete *in vitro* DNA decondensation is critical to increase the sensitivity of methods evaluating its integrity, thus allowing researchers to obtain reliable values of DNA damage. In fact, some authors explained the low sensitivity of techniques assessing DNA integrity through the hypothesis of “the iceberg tip,” which surmises that, while some sperm detected as “normal” actually have DNA breaks, they are out of the method threshold (Gosálvez et al., 2013; Cho et al., 2017).

In the present study, we found that pig and cattle sperm present higher grade of DNA condensation, which is related to an increased difficulty to achieve its complete decondensation *in vitro* and form a sperm halo in an agarose matrix (Figure 2). In pigs, previous studies using the SCD test considered that sperm with non-fragmented DNA lacked a halo, whereas those with fragmented DNA showed a large halo (Enciso et al., 2006; Alkmin et al., 2013; Batista et al., 2016). As shown for pig sperm after 0 min of incubation in the third lysis solution containing proteinase K (Figure 2), the lack of halo identified in other studies (Enciso et al., 2006) could be due to an incomplete DNA decondensation. Therefore, when complete DNA decondensation was achieved following incubation in the third lysis solution containing proteinase K for 180 min, sperm with non-fragmented DNA showed large halos, as occurs when the halo assay is used with human sperm (Fernández et al., 2005). Remarkably, our results showed that complete sperm DNA decondensation is more easily achieved in the human than in the pig. In effect, in order for similar levels of decondensed DNA to be obtained, pig sperm were observed to need a longer lysis treatment with proteinase K. In fact, proteinase K was found to act as a DNA decondensation accelerator, probably because it degrades protamines extracted by the lysis buffer (Figure 4). Previous studies using the SCD test in pig sperm lysed for 5 min, regardless of the composition of solutions (Enciso et al., 2006; Alkmin et al., 2013; Yeste et al., 2013a, b; Batista et al., 2016), are in agreement with our results. In a similar way, using the Comet assay in pig sperm without a long lysis containing proteinase K has also been suggested to lead to sensitivity-compromised results (Pérez-Llano et al., 2010; Enciso et al., 2011). Also matching with our data, a previous study aimed at elucidating alkaline labile sites in pig sperm showed the need of incubating these cells in a lysis solution containing SDS, DTT and proteinase K for 3.5 h at 37°C to observe the same amount of those sites in leukocytes from the same species (Cortés-Gutiérrez et al., 2009). Furthermore, other authors incubated pig sperm with lysis solutions containing proteinase K and found that Comet images depicted complete DNA decondensation (Fraser and Strzezek, 2005).

Regarding the analysis of sperm DNA integrity in cattle, previous studies showed that sperm with non-fragmented DNA depicted small halos, whereas those with fragmented DNA presented big halos, which was in agreement with what found in pigs (García-Macías et al., 2007; Dogan et al., 2015). Our results also indicated that bovine sperm exhibited a small halo when either not exposed or shortly exposed to proteinase K for 30 min. However, when the time of incubation with proteinase K increased, sperm chromatin of bovine sperm completely decondensed, in a similar fashion to what observed in other species (Figure 3). Thus, our results confirm that proteinase K acts as an accelerator of DNA decondensation, and suggest that previous studies evaluating DNA fragmentation in bovine sperm could not have decondensed DNA enough, since the lysis period was, regardless of the solutions used, short (10 min) (García-Macías et al., 2007; Dogan et al., 2015). The use of the Comet assay in cattle sperm also demonstrated that longer lysis treatments increased the degree of DNA decondensation (Villani et al., 2010). However, it remains unclear whether exposure to lysis solutions overnight in Fraser and Strzezek (2005) could have induced artifactual DNA breaks, thus misestimating the actual results.

It is widely known that sperm DNA in humans and horses is condensed around both protamine 1 and protamine 2, whereas only protamine 1 but not protamine 2 is present in the chromatin of pig and cattle sperm (Corzett et al., 2002). Moreover, morphometric studies indicated that pig and cattle sperm display a bigger disk-shaped nucleus, whereas human and horse sperm are smaller and more oval-shaped (Gu et al., 2019). Our results on the resilience of sperm DNA to decondensation in the aforementioned species suggest that while sperm containing only protamine 1 display less efficiency in condensing their DNA, they have stronger protamine-protamine junctions. In fact, protamine 1 in pig sperm displays the highest number of cysteine residues (10 residues) compared to other species, such as donkey (8 residues), horse (7 residues) or human (6 residues), which would support the relevance of disulfide bonds between protamines in the pig (Gosálvez et al., 2011). Furthermore, bovine protamine 1 has less cysteine residues (7 residues) than its pig counterpart, which could explain the less degree of DNA decondensation observed at 0 min of incubation with the third lysis solution (Gosálvez et al., 2011). Alternatively, sperm from species containing both P1 and P2 appear to exhibit higher DNA condensation, with the contraindication of having less protamine-protamine unions, as different protamines from P2 family have less cysteine residues than P1 (Corzett et al., 2002; Oliva, 2006). Moreover, when the number cysteine residues of P1 is related to the P1/P2 ratio, the amount of cysteine residues in sperm chromatin (cysteine residues of P1 × %P1) in each species appears to be linked to the degree of DNA condensation (halo/core ratio) after 0 min of incubation with the third lysis solution (Supplementary Table 1). All the aforementioned could also explain why alterations in the relative amounts of protamine 2 lead to higher sperm DNA decondensation, and underlie DNA damage and infertility (Aoki et al., 2005; García-Peiró et al., 2011; Nanassy et al., 2011).

## Conclusion

In conclusion, long lysis treatment of porcine and bovine sperm is required for complete sperm DNA decondensation in these species. This is not necessary for human, horse and donkey sperm, as lysis solutions used in previous studies led to complete DNA decondensation without any additional treatment. Moreover, we observed that the inclusion of proteinase K accelerates DNA decondensation. Thus, we hypothesize that the species-specific differences in the resilience of sperm DNA to decondensation rely upon whether sperm DNA is condensed around protamine 1 and protamine 2, or around protamine 1 only.

## Data Availability Statement

The original contributions presented in the study are included in the article/Supplementary Material, further inquiries can be directed to the corresponding author/s.

## Ethics Statement

The studies involving human participants were reviewed and approved by the Josep Trueta Hospital Ethics Committee (Reference PTI-HUMA 10012018). The patients/participants provided their written informed consent to participate in this study.

## Author Contributions

JR-M conceived the study, performed the experiments, analyzed the data, run statistics, wrote the manuscript, and approved the final version. EG-B performed the experiments, critically revised the document, and approved the final version. CH, JC, and JM cryopreserved semen samples, critically revised the manuscript, and approved the final document. MY contributed to the experimental design, coordinated the work, discussed results, critically revised the manuscript, and approved the final document. All authors contributed to the article and approved the submitted version.

## Conflict of Interest

The authors declare that the research was conducted in the absence of any commercial or financial relationships that could be construed as a potential conflict of interest.
