# Supplementary material for: Species-Specific Differences in Sperm Chromatin Decondensation Between Eutherian Mammals Underlie Distinct Lysis Requirements
Source: Front Cell Dev Biol. 2021 Apr 30;9:669182. doi: 10.3389/fcell.2021.669182 (PMC8120241; doi:10.3389/fcell.2021.669182)
Supplement: Supplementary file 1 [file Table_1.docx]

**Supplementary table 1**. P1/P2 ratio and number of cysteine residues in each protamine, in relation to our mean DNA decondensation (halo/core at 0 minutes). Mean cysteine content was calculated using P1/P2 ratio and number of cysteine residues in each protamine.

Data was extracted from Gosálvez et al., 2011.

|  | Mean  DNA condensation (halo/core 0 min) | P1/P2 ratio | Number of cysteine residues in P1 | Number of cysteine residues in P2 | Mean Cysteine content |
| --- | --- | --- | --- | --- | --- |
| Human | 4.68 | 0.43 | 6 | 2 | 4.28 |
| Equine | 4.15 | 0.25 | 7 | 2 | 5.75 |
| Donkey | 4.4 | No data available | 8 |  |  |
| Pig | 1.77 | N/A | 10 | 0 | 10 |
| Cattle | 2.4 | N/A | 7 | 0 | 7 |
